# From Innovation to Translation: Emerging Direct‐Analysis Mass Spectrometry Platforms

**DOI:** 10.1002/jms.70125

**Published:** 2026-10-19

**Authors:** Thais Lino, Adriano Reis José da Silva, Davi Rodrigues da Silva, Welisson Vilarindo de Oliveira, Diego Gomes Rocha, Rodinei Augusti

**Affiliations:** ^1^ Mackenzie Institute for Research in Graphene and Nanotechnologies (MackGraphe), School of Engineering, Mackenzie Presbyterian University São Paulo Brazil; ^2^ Scientific Police of the State of Goiás Goiânia Brazil; ^3^ Department of Chemistry Federal University of Minas Gerais Belo Horizonte Brazil; ^4^ Federal Center for Technological Education of Minas Gerais Belo Horizonte Brazil

**Keywords:** ambient ionization mass spectrometry, dielectric‐barrier‐discharge ionization, direct‐analysis mass spectrometry, liquid electron ionization, substrate‐based ionization

## Abstract

Ambient and direct‐analysis mass spectrometry has shifted from predominantly proof‐of‐concept rapid screening to increasingly robust analytical platforms with clearer translational potential. This focused critical review examines the following four emerging or strategically relevant platforms: dielectric‐barrier‐discharge ionization (DBDI), liquid electron ionization (LEI), paper cone spray ionization (PCSI), and sandpaper spray ionization (SPS). DBDI, PCSI, and SPS are ambient or ambient‐like direct‐sampling approaches. LEI is included as a complementary direct‐analysis platform because its electron‐ionization chemistry provides structurally informative fragmentation and can address limitations of soft‐ionization methods. The review emphasizes recent advances in source and substrate engineering, analytical reproducibility, quantitative readiness, portability, and application‐driven implementation. The review uses representative evidence from the literature to distinguish demonstrated analytical performance from broader translational potential, while recognizing that reported detection limits and precision values depend on the method, matrix, and analyte. Rather than cataloging all ambient ionization methodologies, the article critically evaluates the mechanistic strengths, limitations, maturity, and realistic implementation prospects of the four selected platforms in forensic, environmental, pharmaceutical, and field‐analytical contexts. Collectively, these technologies illustrate a broader maturation of direct‐analysis mass spectrometry, and their long‐term impact will increasingly depend on quantitative validation, reproducibility, workflow standardization, and integration with practical analytical needs.

## 1 Introduction

Mass spectrometry (MS) remains one of the most powerful and versatile analytical technologies in modern chemistry, owing to its exceptional sensitivity, molecular specificity, structural elucidation capability, and broad applicability across pharmaceutical analysis, forensic science, clinical diagnostics, environmental monitoring, food chemistry, metabolomics, proteomics, biomolecular research, and materials characterization [1, 2, 3, 4]. Despite these strengths, conventional MS workflows often rely on labor‐intensive sample preparation, solvent‐intensive extraction, chromatographic separations, and centralized instrumentation, which can increase analysis time, reduce throughput, and limit applicability in time‐sensitive, field‐deployable, or in situ analytical scenarios [3].

The emergence of ambient ionization mass spectrometry (AIMS) fundamentally transformed this analytical paradigm by demonstrating that chemically informative mass spectrometric measurements could be obtained directly from untreated or minimally prepared samples under atmospheric conditions [5]. Since the introduction of desorption electrospray ionization (DESI) by Takáts et al. [6] and direct analysis in real time (DART) by Cody et al., [7] ambient ionization approaches have challenged the traditional assumption that meaningful MS analysis necessarily requires extensive sample pretreatment. Instead, solids, liquids, tissues, aerosols, vapors, and complex surfaces can often be interrogated directly in their native or near‐native states, even outside the laboratory environments [2, 3, 8].

Over the past two decades, AIMS has evolved into a diverse field encompassing spray‐based, plasma‐based, thermal, laser‐assisted, and hybrid ionization strategies [2, 9, 10, 11, 12, 13, 14]. Spray‐based methods such as DESI, nano‐DESI, paper spray, and extractive electrospray rely primarily on charged droplets and solvent‐mediated extraction [13, 15]. Plasma‐based techniques such as DART, low‐temperature plasma (LTP), and DBDI use reactive species generated at atmospheric pressure to promote desorption and ionization [2, 16]. Thermal and laser‐assisted approaches, including ASAP (atmospheric solids analysis probe) and LAESI (laser ablation electrospray ionization), provide alternative desorption mechanisms [2, 14].

This methodological diversification has substantially broadened the scope of ambient MS, enabling important applications in food authentication and safety analysis, forensic screening, clinical diagnostics, microbiological differentiation, environmental monitoring, cultural heritage science, pharmaceutical analysis, and biological tissue characterization [17, 18, 19, 20, 21, 22, 23, 24, 25]. More recently, hybrid analytical platforms integrating multiple desorption and ionization mechanisms, engineered substrates, functional sorptive materials, derivatization strategies, and immunoaffinity‐based interfaces have further improved analytical sensitivity, selectivity, and operational robustness [17, 26, 27, 28, 29, 30].

Parallel technological advances have accelerated the field's maturation. Coupling ambient ionization sources with portable, miniaturized mass spectrometers has enabled near‐real‐time molecular analysis outside centralized laboratories, supporting applications in hazardous‐substance detection, border inspection, food‐fraud investigation, field toxicology, and point‐of‐care diagnostics [31]. Similarly, integration with high‐resolution mass analyzers, ion mobility spectrometry, and advanced computational workflows has improved selectivity, structural elucidation, isomer discrimination, and confidence in analyte identification in increasingly complex matrices [32, 33].

Ambient mass spectrometry imaging has also emerged as a major growth area, particularly through DESI‐MSI and nano‐DESI‐MSI, enabling spatially resolved molecular characterization in tissue pathology, intraoperative surgical assessment, plant metabolomics, and biomolecular mapping [34, 35, 36, 37, 38, 39, 40, 41, 42, 43]. In parallel, machine‐learning‐assisted interpretation of high‐throughput spectral datasets has shown increasing promise for tissue classification, counterfeit pharmaceutical detection, food authenticity verification, and anomaly detection [44].

Despite this remarkable expansion, rapid methodological diversification has also introduced conceptual fragmentation. Many reported ambient ionization platforms remain highly specialized proof‐of‐concept demonstrations, whereas relatively few have demonstrated sufficient reproducibility, quantitative robustness, interlaboratory reliability, or translational readiness for broader routine implementation [9, 12, 27, 28, 29, 30, 45, 46, 47, 48]. Persistent challenges—including matrix effects, ion suppression, incomplete mechanistic understanding, heterogeneous sampling behavior, constrained quantitative performance, experimental variability, and limited standardization—continue to restrict widespread adoption [9, 12, 27].

Consequently, the most significant recent progress in ambient and direct‐analysis MS may no longer be defined simply by the proliferation of new ionization acronyms. Instead, progress increasingly reflects source engineering, analytical reliability, quantitative capability, integration with realistic workflows, automation, and practical implementation. Quantitative performance matters because direct‐analysis measurements can be affected by matrix effects, surface heterogeneity, variable extraction efficiency, ion suppression, and sampling geometry. Recent studies show that controlled sampling, optimized source design, internal standards, automated deposition, and chemometric correction can improve precision and quantitative reliability [11, 49]. In this context, the present article does not attempt to provide an exhaustive catalog of ambient ionization methodologies. Instead, it focuses on four emerging or strategically relevant direct‐analysis MS platforms, emphasizing work published from 2021 to 2026: dielectric‐barrier‐discharge ionization (DBDI), liquid electron ionization (LEI), paper cone spray ionization (PCSI), and sandpaper spray ionization (SPS). DBDI, PCSI, and SPS operate within ambient or ambient‐like direct‐sampling workflows. We treat LEI as a complementary direct‐analysis platform rather than a strict ambient ionization method because its hard‐ionization chemistry provides structural information that many soft‐ionization approaches cannot obtain. We selected the four platforms for their mechanistic diversity, recent technological development, demonstrated analytical utility, and relevance to practical workflows. The inclusion of LEI therefore extends the review from a strict taxonomy of ambient ionization to a broader evaluation of direct‐analysis MS technologies that address complementary analytical needs. The comparison is intentionally critical: performance is assessed alongside reproducibility, quantitative evidence, portability, sampling constraints, and translational maturity.

## 2 Dielectric‐Barrier‐Discharge Ionization

DBDI is a plasma‐based ambient ionization strategy that combines rapid direct sampling with broad chemical compatibility [50, 51]. Its source architecture uses an alternating high voltage applied across electrodes separated by a dielectric barrier, generating a nonthermal plasma at atmospheric pressure. The resulting reactive species promote analyte desorption and ionization, while source geometry, gas composition, electrical parameters, and thermal conditions influence analytical performance [50, 52].

Na et al. introduced dielectric barrier discharge ionization (DBDI) as an ambient ionization source in 2007. Their source consisted of a copper sheet electrode, a discharge electrode, and a glass slide that served as both the dielectric barrier and sample plate, on which they generated a stable low‐temperature plasma. The authors demonstrated the source for analyzing 20 amino acids, including L‐alanine. The reported limit of detection (LOD) for L‐alanine was 3.5 pmol using selected‐ion monitoring (SIM), while the relative standard deviation (RSD) was 5.78% for 17.5 nmol of L‐alanine (*n* = 5), demonstrating the quantitative potential of the initial DBDI implementation [53]. A related 2007 study demonstrated the direct detection of TNT, RDX, and PETN on solid surfaces using DBDI‐MS. The reported detection limits were 10 pg for TNT, 0.1 ng for RDX, and 1 ng for PETN. For TNT, an RSD of 5.57% was obtained for 100 pg deposited on different matrices, including paper, cloth, chemical fiber, glass, paint, and soil [54].

The dielectric barrier limits direct electrode contact and can reduce electrode erosion and contamination while stabilizing plasma formation [50, 52]. Depending on operating conditions, the discharge may contain filamentary or more homogeneous microdischarges. Recent work using benzylammonium thermometer ions demonstrated that the geometry of plasma‐based ion sources strongly influences ion heating and in‐source fragmentation. As illustrated in Figure 1, different DBD‐based sources employ distinct plasma configurations, and directing the plasma on‐axis toward the mass‐spectrometer inlet reduced internal energy deposition compared with the conventional orthogonal arrangement, likely by minimizing direct ion interaction with the most energetic region of the plasma jet [50].

**FIGURE 1:**
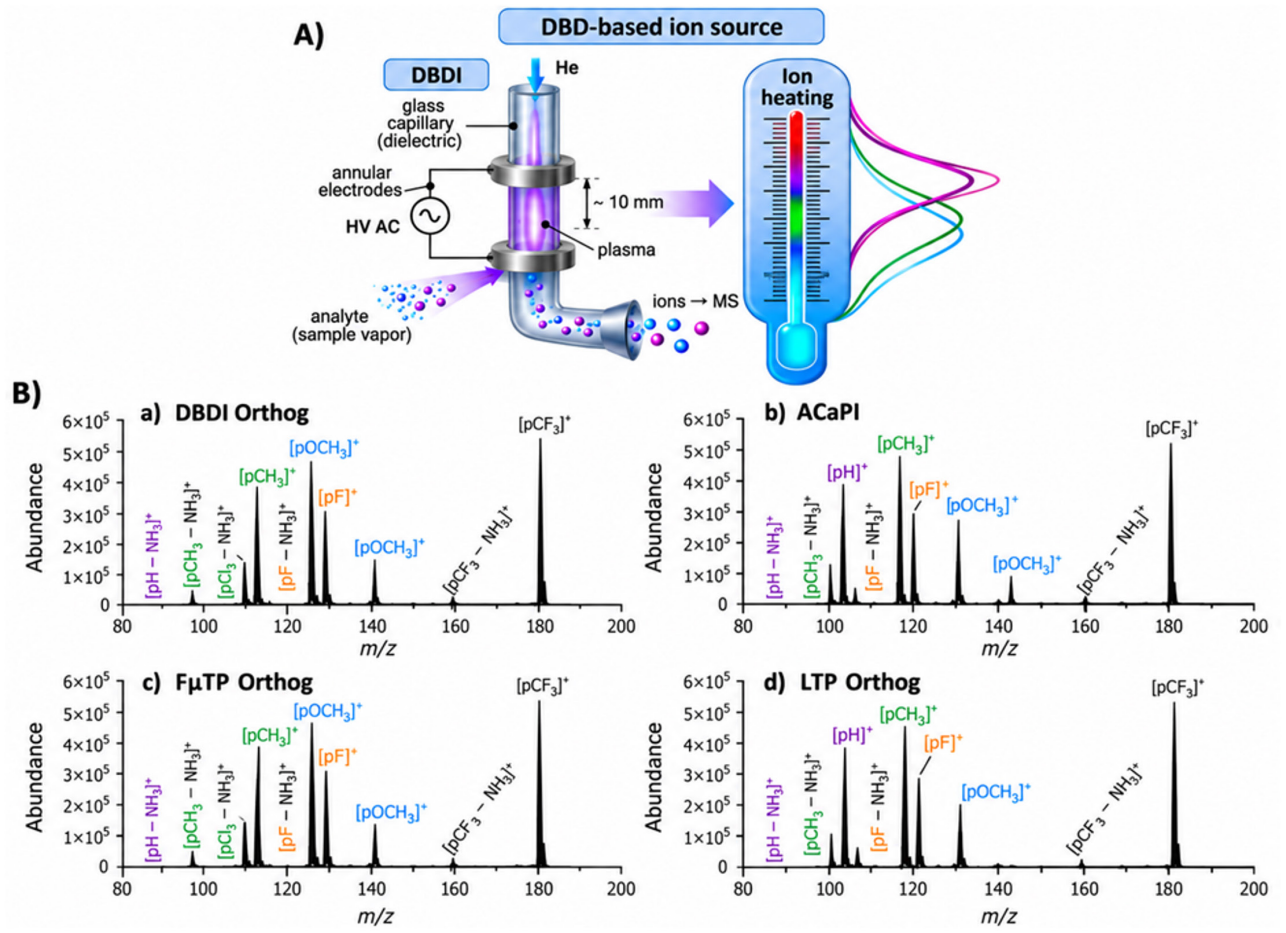
(A) Generic configuration of a dielectric‐barrier‐discharge ionization (DBDI) source. An alternating high voltage is applied across electrodes separated by a dielectric barrier, generating nonthermal plasma that promotes analyte desorption and ionization near the mass‐spectrometer inlet. DBDI implementations vary in geometry, gas supply, and electrode arrangement. (B) Representative mass spectra of a mixture of the following five benzylammonium thermometer ions obtained using different dielectric barrier discharge (DBD)‐based ion sources: (a) dielectric barrier discharge ionization (DBDI) in the orthogonal configuration, (b) active capillary plasma ionization (ACaPI), (c) flexible microtube plasma (FμTP) in the orthogonal configuration, and (d) low‐temperature plasma (LTP) in the orthogonal configuration. The intact benzylammonium precursor ions and their corresponding product ions formed by fragmentation are indicated. Adapted with permission from reference 50, licensed under CC BY 4.0.

These plasma conditions generate ions, radicals, metastable species, and excited neutrals that can promote desorption, excitation, dissociation, and ionization. This flexibility helps DBDI accommodate polar and less‐polar compounds, volatile species, and diverse matrices, but it also makes source optimization important for reproducibility [50, 51].

A major practical advantage of DBDI is its broad ionization versatility. Heat‐assisted DBDI can increase volatilization, desorption, transport, and signal intensity, which is useful for viscous or low‐volatility samples but may compromise thermally labile analytes if heating is excessive [51]. In a 2025 headspace‐injection study, optimized DBDI achieved detection limits from the high‐nanomolar to low‐micromolar range for tested compounds. For ethanol, the optimized method gave a 0.1 mM LOD, while repetitive injections showed an RSD of 5.6% at 10 mM; selected VOCs showed LODs of 0.00025–0.005 mM [55].

DBDI has demonstrated applicability in food analysis, forensic investigations, biomarker detection, clinical monitoring, and volatile organic compound profiling [51, 55]. These applications illustrate its value for rapid screening and monitoring, not that DBDI replaces chromatographic workflows. Where DBDI is coupled to chromatographic systems, the resulting configurations are better considered hybrid extensions of the source technology.

From a translational perspective, DBDI combines relatively simple source architecture, atmospheric‐pressure operation, rapid direct analysis, reduced sample preparation, and the option of assisted heating [50, 52, 55, 56]. Its quantitative record is promising, but the reported figures remain strongly method‐dependent and do not by themselves establish interlaboratory comparability. However, important limitations remain. Reproducibility depends on source geometry, operating conditions, sampling configuration, and matrix composition, while mechanistic complexity can complicate standardization. The diversity of source designs also limits commercial maturation. Broader implementation will require standardized operating protocols, interlaboratory benchmarking, and validation across representative matrices. Overall, DBDI is a comparatively mature plasma‐based direct‐analysis platform with broad analyte compatibility and demonstrated quantitative potential. Its principal translational challenge is converting source flexibility into standardized, reproducible analytical performance.

## 3 Liquid Electron Ionization

Liquid electron ionization mass spectrometry (LEI‐MS) is a complementary direct‐analysis platform that extends electron ionization to liquid‐phase workflows. By converting a liquid stream into the gas phase before EI (electron ionization), LEI enables structurally informative fragmentation for compounds that can be difficult to characterize by soft ionization [57, 58, 59].

LEI extends EI beyond traditional gas chromatography by converting the chromatographic effluent into gas‐phase analytes before ionization, enabling analysis of liquid‐phase samples and overcoming the intrinsic incompatibility between liquid flow and high‐vacuum EI sources [57, 58]. This innovation significantly expands the applicability of EI‐based mass spectrometry beyond conventional gas chromatographic workflows. It is particularly advantageous for analyzing nonpolar, isomeric, and small‐ to medium‐molecular‐weight compounds that are often difficult to characterize effectively with electrospray‐based ionization methods [57, 58, 59].

A major analytical strength of LEI‐MS is its ability to generate highly reproducible, structurally informative fragmentation patterns characteristic of EI, enabling reliable compound identification through mass spectral matching against well‐established libraries such as NIST (National Institute of Standards and Technology). Compared with many soft‐ionization direct‐analysis approaches, LEI also shows reduced susceptibility to matrix effects, improving analytical robustness and quantitative reliability in chemically complex samples [57, 58]. Compatibility with both low‐ and high‐resolution mass analyzers further broadens its utility across targeted, untargeted, qualitative, and semiquantitative analytical workflows [57, 59, 60].

Recent technological developments in interface engineering, including improvements in vaporization microchannels, capillary design, flow control, and analyte transfer efficiency, have significantly enhanced sensitivity, vaporization performance, and overall analytical robustness [58, 59]. These refinements have strengthened compatibility with modern nanoflow liquid chromatography systems and expanded applicability in challenging analytical scenarios such as forensic investigations, toxicological analysis, pharmaceutical characterization, and environmental monitoring [57, 59].

LEI also has limitations. EI can produce extensive fragmentation and reduce molecular‐ion abundance, which may be disadvantageous when intact molecular ions are required. The interface is more complex and less portable than many ambient sources. Nevertheless, these trade‐offs can be advantageous when reproducible EI spectra, library matching, and structural discrimination are the primary objectives [57, 58, 59].

An important recent extension of this concept is extractive‐liquid sampling electron ionization mass spectrometry (E‐LEI‐MS), which helps bridge the conceptual gap between ambient‐like direct sampling and structurally rich EI‐based analysis [61]. In this approach, analytes are extracted directly from sample surfaces under ambient conditions using microliter volumes of solvent and then introduced into an EI source, where rapid vaporization and gas‐phase ionization occur (Figure 2A) [61]. Unlike ambient soft‐ionization techniques such as ESI or DESI, E‐LEI‐MS produces highly reproducible fragmentation patterns that support confident compound identification through established NIST spectral databases (Figure 2B) [61].

**FIGURE 2:**
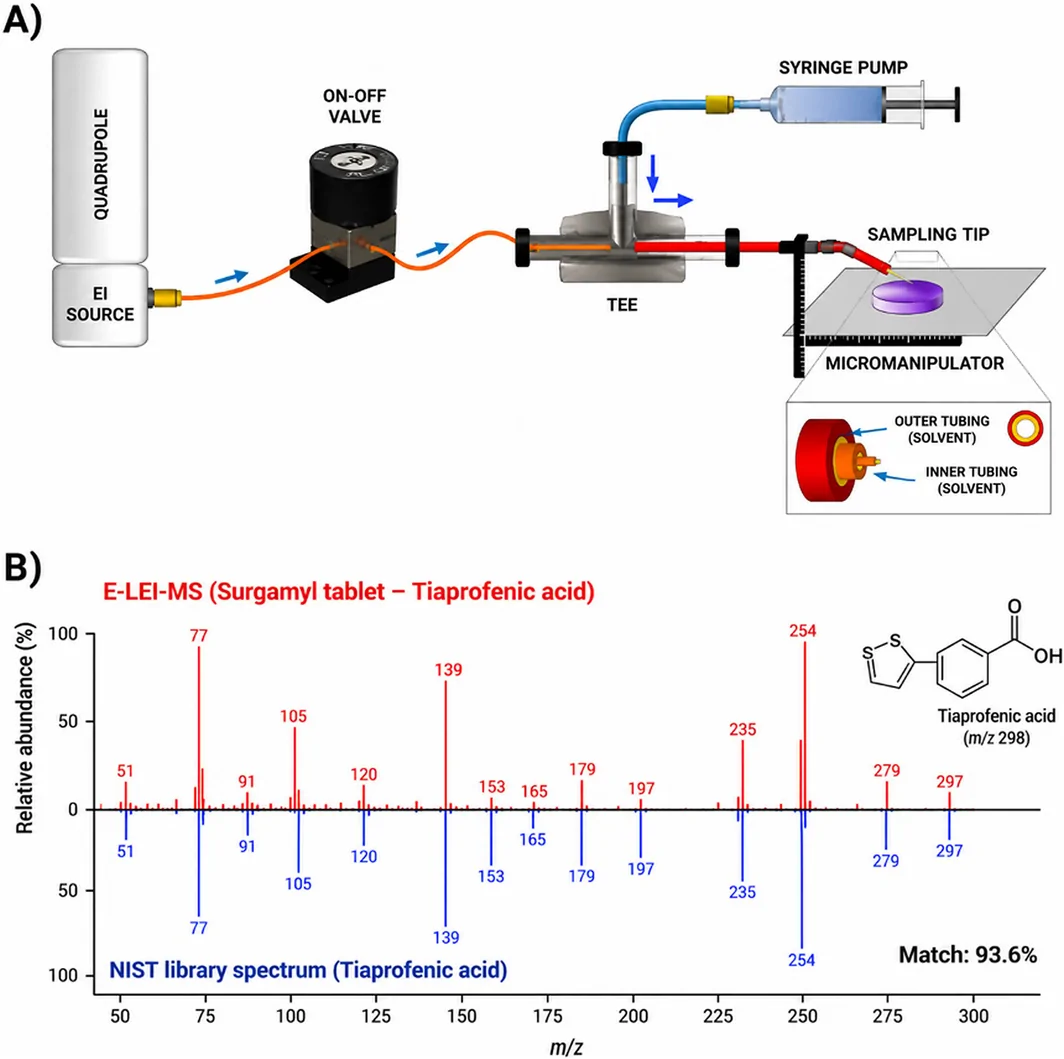
(A) Configuration of the extractive‐liquid sampling electron ionization‐mass spectrometry (E‐LEI‐MS) system. (B) Representative E‐LEI‐MS spectrum of tiaprofenic acid obtained from the direct analysis of a Surgamyl tablet, showing the experimental EI mass spectrum and its comparison with the NIST library spectrum. Adapted from reference 61, licensed under CC BY 4.0.

E‐LEI‐MS offers the practical advantage of rapid targeted and untargeted analysis without chromatographic separation or extensive sample preparation, substantially reducing analytical turnaround time [61, 62]. Applications have demonstrated their utility in forensic and pharmaceutical contexts requiring fast, reliable screening, including direct detection of active pharmaceutical ingredients, excipients, illicit substances, and surface residues in complex matrices [62]. The successful identification of benzodiazepines and other drugs from trace surface residues further highlights their relevance in realistic forensic scenarios, including investigations involving drug‐facilitated crimes [62]. Overall, LEI provides a complementary direct‐analysis capability: it prioritizes reproducible structural information and spectral‐library compatibility over the minimal instrumentation and soft‐ionization behavior characteristic of many ambient sources.

## 4 Paper‐Spray‐Derived and Substrate‐Based Platforms: PCSI and SPS

### 4.1 Paper Cone Spray Ionization

Paper cone spray ionization (PCSI) represents an important maturation of substrate‐based direct‐analysis mass spectrometry [63]. Whereas conventional paper spray ionization established the feasibility of integrating rapid sampling, analyte extraction, and direct mass spectrometric analysis into a simple analytical workflow, PCSI—particularly in its three‐dimensional‐printed implementations—introduces meaningful engineering refinements that substantially improve analytical reproducibility, mechanical robustness, geometric control, and design customizability [20, 64, 65].

Among the most significant recent developments is 3D‐printed PCSI (3D‐PCSI‐MS), which integrates additive manufacturing with direct sampling and electrospray‐like ionization using conductive polymeric cones [64, 66]. Compared with manually fabricated paper devices, controlled printing improves geometric reproducibility, mechanical robustness, and manufacturing consistency. The approach also integrates sampling, extraction, and ionization into a single analytical platform, simplifying sample handling and improving practical reliability for rapid direct analysis. Solid or particulate samples are introduced directly into the cone cavity, then a suitable solvent is added to promote in situ analyte extraction. When high voltage is applied, the cone tip generates spray ionization through a mechanism analogous to electrospray or conventional paper spray ionization (Figure 3A) [20, 64, 65]. As a representative application of the 3D‐PCSI‐MS platform, Figure 3B presents the MS/MS spectrum of [TATP + Na]^+^, demonstrating the detection of the explosive triacetone triperoxide (TATP) in a postblast sample [20].

**FIGURE 3:**
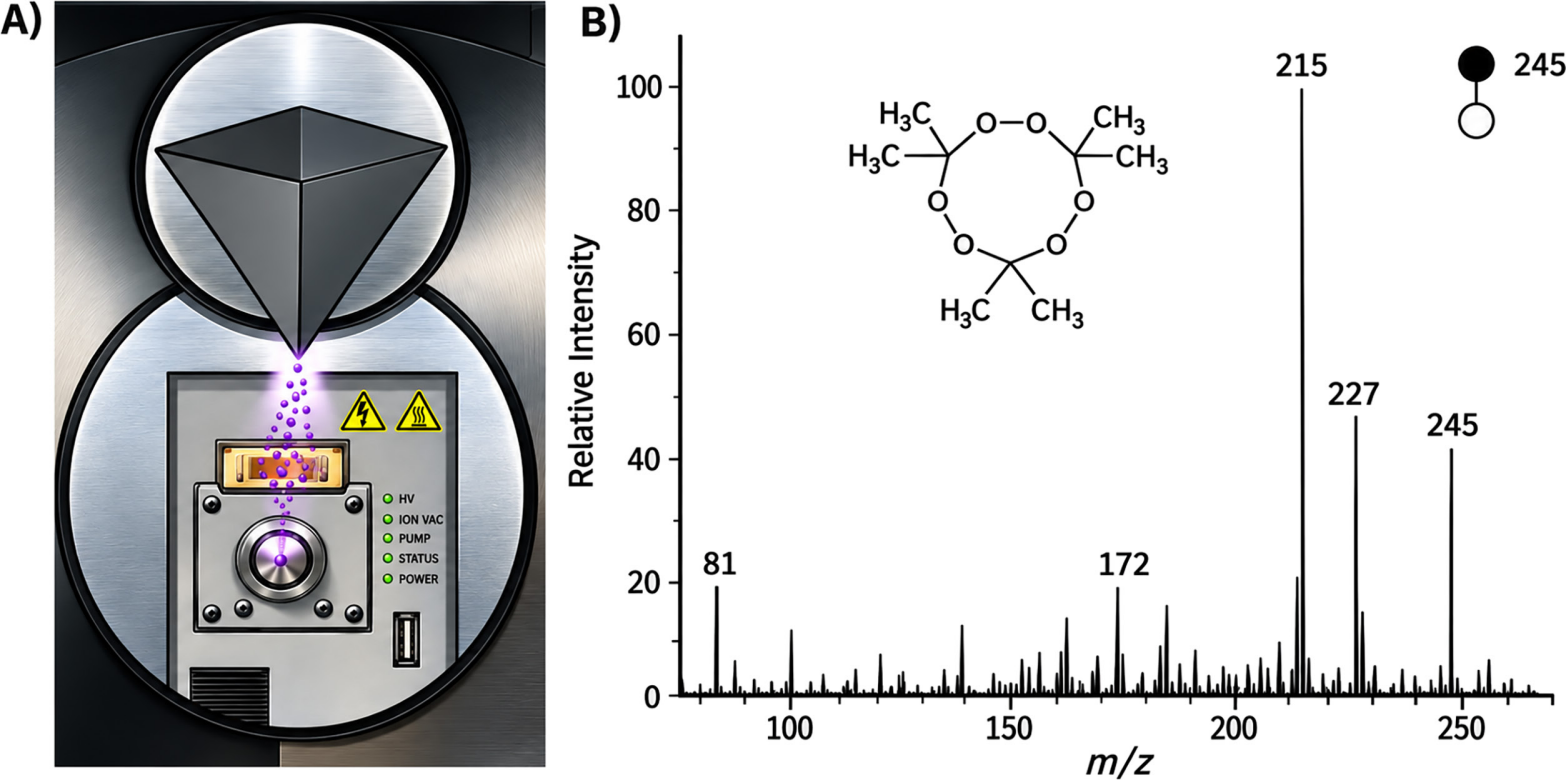
(A) Schematic representation of the three‐dimensional printed cone spray ionization mass spectrometry (3D‐PCSI‐MS) platform. (B) Tandem mass spectrum (MS/MS) of triacetone triperoxide (TATP) detected in a sample collected after an explosive event (postblast sample), observed as its sodium adduct, [TATP + Na]^+^. Adapted with permission from reference 20, licensed under CC BY 4.0.

A notable strength of PCSI lies in its adaptability to diverse analytical scenarios through material and design customization. Device construction materials must be selected according to solvent compatibility and analytical objectives. For example, polyvinylidene fluoride (PVDF) offers broad chemical resistance. Still, it may be unsuitable for fluorinated analytes such as per‐ and polyfluoroalkyl substances (PFAS), whereas PETG (polyethylene terephthalate glycol) and thermoplastic copolyester elastomers provide cost‐effective alternatives with improved compatibility in certain applications [64].

PCSI has demonstrated rapid analysis of solid and particulate matrices with minimal preparation [20, 64]. In a study of PFAS (polyfluoroalkyl substances) in six soil and sediment matrices, 3D‐PCSI‐MS identified them using diagnostic MS^3^ spectra, with reported LODs of 100 ppt to 10 ppb depending on the PFAS and soil classification. The sampling rate was below 2 min per sample, supporting high‐throughput screening of contaminated areas [66]. These values provide a concrete example of quantitative sensitivity, but they should not be interpreted as directly comparable with DBDI or LEI because the analytes, matrices, instruments, and validation designs differ.

From a translational perspective, PCSI aligns closely with broader trends in ambient mass spectrometry toward rapid analysis, reduced sample handling, automation compatibility, and field‐deployable analytical workflows [19, 20, 21, 27, 67]. Its compatibility with portable mass spectrometric systems is particularly important, as it strengthens its potential for on‐site environmental screening, forensic deployment, and other operational scenarios requiring rapid decision‐making [64, 68]. The capacity to accommodate irregular sample surfaces and customized sampling strategies further enhances its practical flexibility [21, 67].

Despite these advantages, important limitations remain. Analytical performance may be significantly influenced by matrix composition, particularly in samples containing high organic content, clay, or isobaric interferents, which can suppress ionization and compromise detection sensitivity [66, 68]. Solvent compatibility also remains an important practical consideration, as aggressive solvents may deform or degrade polymer‐based analytical devices depending on the construction material [64, 66, 68]. Broader translational adoption will also require more extensive interlaboratory validation, improved quantitative benchmarking, and deeper assessment of analytical reproducibility across diverse sample types and operating conditions.

Overall, PCSI currently stands among the most promising translational substrate‐based direct‐analysis mass spectrometric platforms. By integrating customizable engineering, simplified analytical workflows, and compatibility with portable instrumentation, it offers a practical, highly adaptable strategy for rapid, direct analysis of chemical and operationally challenging samples [20, 65, 66, 69].

### 4.2 Sandpaper Spray Ionization

Sandpaper spray ionization mass spectrometry (SPS‐MS) is a specialized evolution of substrate‐based ambient ionization designed to address a specific operational limitation in direct‐analysis mass spectrometry: efficiently interrogating mechanically resistant solid surfaces that are often poorly compatible with conventional substrate‐based approaches [70]. Conceptually derived from paper spray ionization mass spectrometry (PS‐MS), originally introduced by Cooks and colleagues in 2010 [71]. SPS replaces delicate porous sampling substrates with abrasive, high‐surface‐area materials that mechanically transfer analytes directly from solid surfaces while preserving the operational simplicity of electrospray‐like ambient analysis (Figure 4A) [70, 72, 73].

**FIGURE 4:**
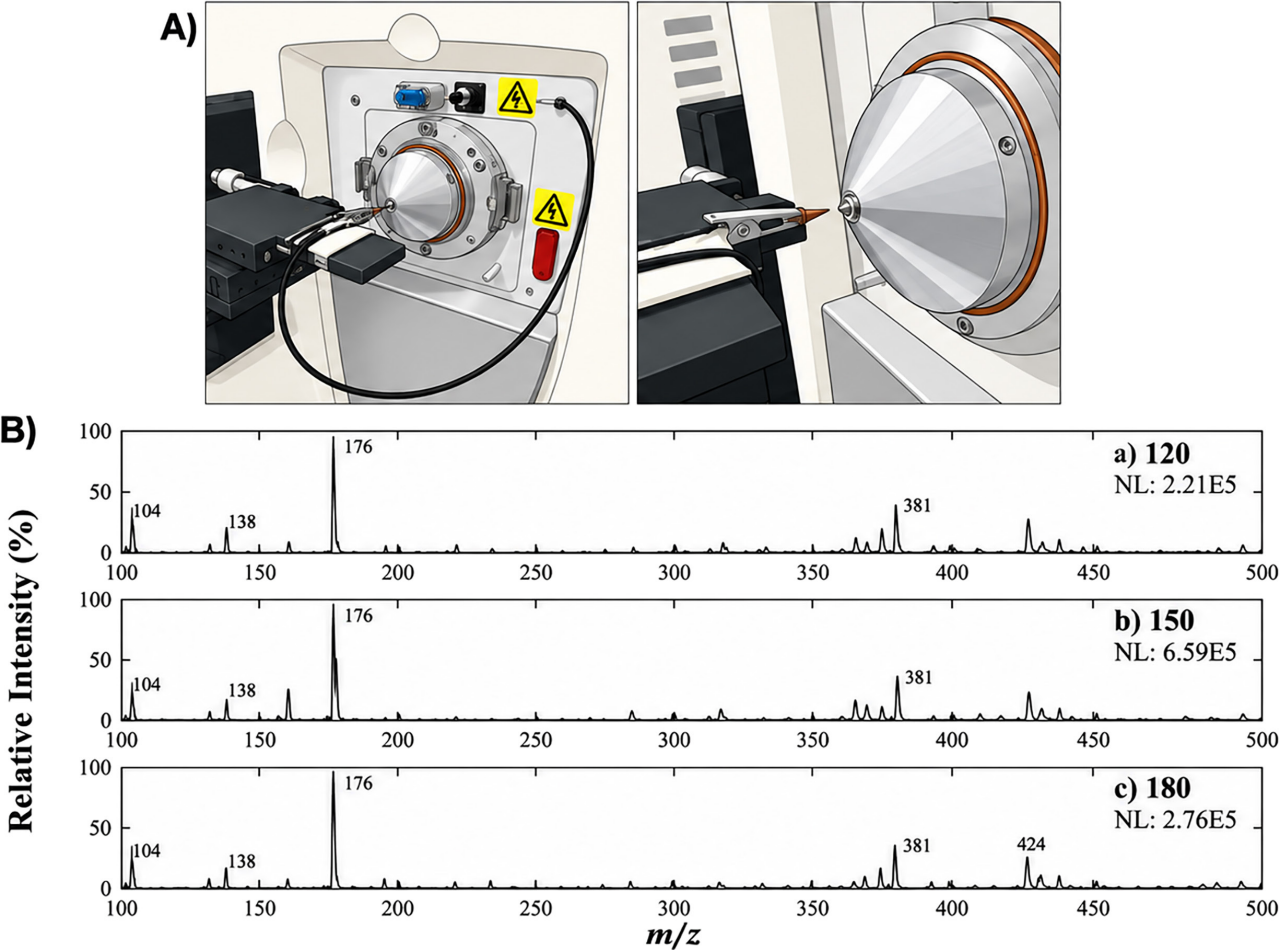
(A) Triangular sandpaper substrate positioned in front of the mass spectrometer inlet for sandpaper spray mass spectrometry (SPS‐MS) analysis. (B) Positive‐ion‐mode SPS mass spectra of green coffee beans obtained using sandpapers with different granulometries: (a) 120, (b) 150, and (c) 180. The NL value represents the sum of the absolute ion intensities in each mass spectrum. Adapted with permission from reference 70, licensed under CC BY 4.0.

The original SPS‐MS proof‐of‐concept, introduced in 2023, employed commercial sandpaper as both a sampling and ionization substrate [70]. In this configuration, analytes were mechanically transferred from hard solid samples via abrasive surface contact, after which the sandpaper was cut into a triangular geometry similar to conventional PS‐MS substrates. Solvent addition, typically methanol, followed by high‐voltage application, generated electrospray‐like ionization from the abrasive substrate [70]. This relatively simple modification substantially expanded the applicability of substrate‐based ambient ionization to materials that are difficult to section, deform, or directly sample using traditional paper‐based approaches (Figure 4B).

A principal strength of SPS is its ability to sample mechanically resistant surfaces with minimal preparation. In the original demonstration, coffee‐bean surfaces were sanded onto triangular sandpaper, enabling detection of caffeine, sugars, and carboxylic acids [70]. The practical advantage over conventional PS‐MS is therefore primarily sampling: the abrasive substrate can transfer analytes from polishable surfaces that are difficult to cut or otherwise conform to a paper‐spray geometry. The study was a proof of principle rather than a validated quantitative comparison.

Subsequent studies expanded SPS into forensic, pharmaceutical, and biological applications [72, 73]. In pharmaceutical tablets and edible cannabis products, SPS‐MS enabled rapid detection of active ingredients and several excipients [73]. Importantly, SPS detected oligosaccharides even though conventional PS‐MS does not readily detect them because hydrophilic sugars can be retained by cellulose‐based paper substrates [73]. SPS has also demonstrated utility in biological classification workflows. Applying SPS to maple (*Acer* sp.) leaves infected with fungal pathogens produced reproducible metabolic fingerprints that distinguished healthy from infected tissues [72]. Integration with chemometric methods, including principal component analysis (PCA) and hierarchical cluster analysis (HCA), enabled clear multivariate discrimination among biological sample groups, emphasizing SPS's suitability for classification‐oriented metabolomic workflows focused on molecular fingerprinting rather than exhaustive biomarker identification [72].

From a translational perspective, SPS offers several attractive features, including low‐cost substrate implementation, operational simplicity, rapid direct analysis, compatibility with chemometric workflows, and strong conceptual potential for integration with portable mass spectrometric systems [70, 72, 73]. These characteristics make the technique particularly appealing for rapid forensic screening, counterfeit pharmaceutical detection, field‐deployable solid‐sample analysis, and classification‐driven analytical applications.

Nevertheless, important limitations remain. SPS is inherently destructive because sampling relies on abrasive mechanical transfer from the sample surface [70]. Because analysis is restricted primarily to surface‐accessible material, compositional representativeness may be limited, particularly for heterogeneous matrices such as pharmaceutical tablets [73]. Additional concerns include potential substrate‐derived background interference depending on the sandpaper composition, significant susceptibility to matrix effects due to the lack of chromatographic separation, and the currently limited breadth of quantitative and interlaboratory validation [70, 72, 73]. Overall, SPS is a pragmatic and specialized extension of paper‐spray analysis. Its strongest current contribution is mechanical sampling of resistant solid surfaces rather than demonstrated quantitative superiority. The technique remains early in validation, with destructive sampling, surface‐limited representativeness, substrate background, and limited quantitative/interlaboratory evidence constraining broader adoption [70, 72, 73].

## 5 Comparative Overview of Emerging Direct‐Analysis Mass Spectrometric Platforms and Quantitative Performance and Cross‐Platform Interpretation

To clarify the AIMS techniques discussed above, Table 1 presents a systematic comparative summary of each method's key advantages, limitations, and potential shortcomings. Table 2 shows some quantitative performance reported for the reviewed platforms. The literature suggests that quantitative readiness is improving, but LOD, LOQ, RSD, and linear‐range values must be interpreted within the analytical context in which they were obtained. A common validation framework across DBDI, LEI, PCSI, and SPS is still lacking, which is an important translational gap.

**TABLE 1:** Comparative assessment of emerging direct‐analysis mass spectrometry platforms, highlighting their analytical features, strengths, limitations, and translational maturity.

AIMS technique	Dominant ionization mechanism	Typical sample types	Quantitative readiness	Portability potential	Principal strengths	Principal limitations	Translational maturity
**DBDI** [50, 51]	Plasma‐based atmospheric ionization	VOCs, liquids, food matrices, pharmaceuticals, residues	Moderate; representative LOD/RSD evidence reported	Moderate to high	Broad analyte compatibility, minimal preparation, low‐cost construction	Optimization sensitivity, matrix dependence	Moderate
**LEI** [61]	Electron ionization after liquid‐to‐gas conversion	Pharmaceutical, forensic residues, and environmental samples	Moderate to high	Low to moderate	Structural specificity, reproducible fragmentation, and spectral library compatibility	Interface complexity; limited portability	Moderate
**PCSI** [65]	Electrospray‐like cone spray ionization	Soils, sediments, explosives, PFAS, forensic solids	Moderate; low‐LOD screening demonstrated	Moderate to high	Integrated sampling/extraction/ionization, customizable geometry	Matrix suppression, solvent‐material compatibility	Moderate to high
**SPS** [70]	Abrasive substrate‐assisted electrospray‐like ionization	Tablets, plant tissues, hard solids	Low to moderate; quantitative validation limited	High potential	Efficient hard‐surface sampling, low‐cost implementation	Destructive sampling, immature validation	Early to moderate

**TABLE 2:** Representative quantitative performance reported for the reviewed platforms. Values are illustrative rather than directly comparable because analytes, matrices, source configurations, analyzers, and validation designs differ.

Platform	Representative application	Metric	Reported value	Interpretive limitation
DBDI [54, 55]	VOCs; early amino acid/explosive studies	LOD; RSD	0.1 mM ethanol; 0.00025–0.005 mM selected VOCs; 5.6% RSD (10 mM ethanol); 5.78% RSD for L‐alanine	Method‐, matrix‐, and source‐dependent; no common interlaboratory benchmark
LEI [58]	Pesticides in soil; LC‐LEI‐MS	LOD	Low‐picogram range for five pesticides	Exact values and validation conditions are compound‐ and method‐specific
PCSI [66]	PFAS in soils/sediments	LOD; throughput	100 ppt–10 ppb; < 2 min/sample	Screening study; values depend on PFAS and matrix
SPS [73]	Pharmaceutical tablets; edible cannabis	Quantitative validation	No LOD/LOQ/RSD reported; qualitative detection demonstrated	Current evidence supports rapid screening, not validated quantitation

## 6 Future Perspectives

Forthcoming progress should prioritize validation frameworks that allow meaningful comparison of direct‐analysis platforms across laboratories, matrices, and instrument configurations. In particular, standardized reporting of LOD, LOQ, precision, linearity, matrix effects, sampling variability, and failure modes would make claims of quantitative readiness more transparent and reproducible. Quantitative maturation should be accompanied by better control of sampling geometry, internal‐standard strategies, automated deposition or collection, and computational correction. Such measures can reduce operator dependence and help translate proof‐of‐concept sensitivity into reproducible measurements.

Commercial development should focus on standardized hardware, interchangeable source components, reproducible substrate manufacturing, simplified user interfaces, and compatibility with portable mass analyzers. For substrate‐based platforms, treat solvent compatibility, substrate background, durability, and manufacturing traceability as design criteria, not secondary considerations. Automation can improve positioning, sample handling, acquisition, and quality control. Robotic or semiautomated workflows could be particularly valuable in forensic screening, pharmaceutical quality control, and environmental surveillance, where throughput and reproducibility matter.

Advanced computational analysis may further increase the value of high‐dimensional direct‐analysis spectra, particularly for classification and anomaly detection. However, deployment in regulated settings will require representative training sets, external validation, transparent performance metrics, and appropriate control of model drift. Portability will remain an important development target. Compact mass analyzers, low‐power ion sources, robust interfaces, and simplified sampling could expand direct‐analysis MS into decentralized settings. Still, field deployment should include quality‐assurance procedures that define when a rapid result is sufficient and when confirmatory laboratory analysis is required.

Future studies should distinguish exploratory demonstrations from validated analytical methods. Clear reporting of the intended decision use, sampling limitations, quantitative validation status, and transferability between instruments will help the field identify which platforms are ready for routine deployment and which remain primarily research tools. The next phase of direct‐analysis mass spectrometry will therefore depend on consolidation rather than proliferation: platforms that combine reproducible sampling, quantitative reliability, operational robustness, and credible validation are most likely to achieve sustained adoption.

Finally, the future relevance of these emerging platforms will depend less on continued methodological proliferation and more on disciplined analytical maturation. Technologies that demonstrate reproducibility, quantitative reliability, interoperability, and practical implementation readiness are most likely to achieve durable impact. In this context, the next defining phase of direct‐analysis mass spectrometry may be characterized not by the rapid expansion of experimental ionization concepts, but by the selective consolidation of analytically credible technologies capable of broader real‐world adoption.

## 7 Conclusions

The reviewed literature indicates a shift in direct‐analysis mass spectrometry from proof‐of‐concept development toward greater analytical credibility, with increasing emphasis on reproducibility, quantitative evidence, engineering refinement, and compatibility with realistic workflows. The four platforms examined illustrate complementary approaches to persistent analytical challenges. DBDI offers broad analyte compatibility and operational simplicity, with demonstrated quantitative performance, but remains sensitive to source and matrix conditions and requires further standardization. LEI provides reproducible EI fragmentation and strong structural specificity, with reduced matrix sensitivity, although its greater interface complexity limits portability. PCSI shows how additive manufacturing can improve sampling reproducibility, mechanical robustness, and field compatibility, while SPS addresses the specific challenge of sampling resistant solid surfaces through a simple mechanical adaptation, although the approach remains comparatively immature.

Collectively, these platforms demonstrate that progress in direct‐analysis MS increasingly depends on improving analytical performance and integration into practical workflows rather than introducing new ionization concepts alone. The quantitative results, summarized in Table 2, also highlight the need for caution when comparing detection limits and precision, as performance depends strongly on the analyte, matrix, instrument, and sampling configuration. Direct‐analysis MS should therefore be regarded as complementary to, rather than a universal replacement for, conventional chromatographic workflows. Its principal advantages lie in applications requiring rapid screening, minimal sample preparation, direct surface analysis, portability, or molecular fingerprinting. Wider routine adoption will require standardized methods, interlaboratory validation, quantitative benchmarking, and robust instrumentation.

## Funding

This work was supported by the Conselho Nacional de Desenvolvimento Científico e Tecnológico, the Coordenação de Aperfeiçoamento de Pessoal de Nível Superior, and the Fundação de Amparo à Pesquisa do Estado de Minas Gerais.

## Disclosure

During preparation of this revised manuscript, we used generative AI‐assisted tools (Grammarly v.1.2.291.1949) exclusively to support language editing. The authors reviewed and verified the scientific content, citations, and final text and remain fully responsible for the manuscript's accuracy, integrity, and originality.

## Data Availability

The data that support the findings of this study are available from the corresponding author upon reasonable request.
